# Protective Effect of Minocycline Hydrochloride on the Mouse Embryonic Development Against Suboptimal Environment

**DOI:** 10.3389/fcell.2022.799042

**Published:** 2022-02-01

**Authors:** Xiaojing Hou, Changming Cai, Yuanlin He, Shiyu An, Shuqin Zhao, Hao Sun, Yang Yang

**Affiliations:** ^1^ State Key Laboratory of Reproductive Medicine, Women’s Hospital of Nanjing Medical University, Nanjing Maternity and Child Health Care Hospital, Nanjing, China; ^2^ State Key Laboratory of Reproductive Medicine, Nanjing Medical University, Nanjing, China

**Keywords:** minocycline hydrochloride, PARP1, mouse embryo, suboptimal environment, ROS

## Abstract

Numerous studies have reported how inner cell mass (ICM) and trophectoderm (TE) was determined during the process of early mouse embryonic development from zygotes into organized blastocysts, however, multiple mysteries still remain. It is noteworthy that pluripotent stem cells (PSCs), which are derived from embryos at different developmental stages, have identical developmental potential and molecular characteristics to their counterpart embryos. Advances of PSCs research may provide us a distinctive perspective of deciphering embryonic development mechanism. Minocycline hydrochloride (MiH), a critical component for maintaining medium of novel type of extended pluripotent stem cells, which possesses developmental potential similar to both ICM and TE, can be substituted with genetic disruption of *Parp1* in our previous study. Though *Parp1*-deficient mouse ESCs are more susceptible to differentiate into trophoblast derivatives, what role of MiH plays in mouse preimplantation embryonic development is still a subject of concern. Here, by incubating mouse zygotes in a medium containing MiH till 100 h after fertilization, we found that MiH could slow down embryonic developmental kinetics during cleavage stage without impairing blastocyst formation potential. Olaparib and Talazoparib, two FDA approved PARP1 inhibitors, exhibited similar effects on mouse embryos, indicating the aforementioned effects of MiH were through inhibiting of PARP1. Besides, we showed an embryonic protective role of MiH against suboptimal environment including long term exposure to external environment and H2O2 treatment, which could mimic inevitable manipulation during embryo culture procedures in clinical IVF laboratory. To our knowledge, it is not only for the first time to study MiH in the field of embryo development, but also for the first time to propose MiH as a protective supplement for embryo culture, giving the way to more studies on exploring the multiple molecular mechanisms on embryonic development that might be useful in assisted reproductive technology.

## Introduction

During early murine embryo development, the zygote undergoes a serials of cell divisions and generates a blastocyst which consists of two distinct cell types: the inner cell mass (ICM) and the trophectoderm (TE) that surrounds the ICM (Johnson and Ziomek 1981; Fleming 1987; Morris et al., 2010). The TE gives rise to trophoblast lineages of placenta, and the ICM forms pluripotent epiblast, which following develops to the embryo proper and the primitive endoderm (Chazaud et al., 2006). Though numerous studies have reported how these lineages are determined, multiple mysteries still remain (Chazaud and Yamanaka 2016).

Studies focused on mechanical regulation of stem cells pluripotency have offered many clues for embryonic development research (Nichols and Smith 2012; Boroviak et al., 2014; Baker and Pera 2018). In our previous study, extended pluripotent stem cells (EPS) were derived from blastocyst and exhibited widespread bi-potency for both embryonic and extraembryonic lineages *in vivo*. This novel type of pluripotent stem cells (PSCs) was maintained with a chemical cocktail, consisting of human leukemia inhibitory factor (hLIF), CHIR 99021, (S)-(+)-dimethindene maleate (DiM) and minocycline hydrochloride (MiH), which was short for LCDM (Yang et al., 2017). Notably, *Parp1*-deficient mouse EPS cultured in condition without MiH could develop into both TE and ICM of the embryos as well (Yang et al., 2017), indicating MiH performed through inhibiting Poly (ADP-ribose) polymerase-1 (PARP1), which was consistent with the work of Alano et al., 2006. What role MiH plays in the embryonic development procedure and whether it works through PARP1 inhibition are subjects of concern.

PARP1 is the most abundant isoform of PARP family. To date, three functionally defined domains of PARP1 has been found: 1) N-terminal DNA binding domain; 2) C-terminal catalytic domain and 3) central automodification domain (Rolli et al., 1997). It is an important nuclear factor in modulating cell mitosis, DNA replication, transcription, metabolism and epigenetic events through PARylation of downstream proteins (Pleschke et al., 2000; Simbulan-Rosenthal et al., 2001; Hassa and Hottiger 2002; Simbulan-Rosenthal et al., 2003; Kanai et al., 2007; Nusinow et al., 2007; Caiafa et al., 2009). Moreover, PARP1 acts as a DNA damage sensor and binds to both single and double stranded DNA breaks (Virag and Szabo 2002; Gibson and Kraus 2012), promoting both base excision repair and homologous recombination (Burkle 2001; Curtin 2013). Studies in somatic cells indicate that when PARP1 activated, it attaches PAR units which were derived from NAD^+^ to various nuclear proteins including itself, histones and other nuclear proteins such as transcription factors (Cohen-Armon 2007; Hinz et al., 2010; Kraus and Hottiger 2013). Several works have proved that genetic and pharmaceutical disruption of PARP1 in oxidative stress played a protective role against cell death by maintaining integrity of mitochondrial membrane and activating phosphatidylinositol-3 kinase (PI3K)-AKT signal pathway (Veres et al., 2004; Tapodi et al., 2005; Bartha et al., 2009; Mester et al., 2009; Szanto et al., 2009).

Oxidative stress, a cellular condition caused by the accumulation of reactive oxygen species (ROS), have been repeatedly shown to be prevalent in defective embryo development and result in suboptimal pregnancy rates (Guerin et al., 2001; Kovacic and Vlaisavljevic 2008; Ciray et al., 2009; Waldenstrom et al., 2009). Although numerous studies have reported the effects of individual antioxidants on embryo development (Fujitani et al., 1997; Ali et al., 2003; Kitagawa et al., 2004; Choe et al., 2010; Silva et al., 2015), it seems that none of the available ones can fully mimic the physiological conditions of the female tract (Aviles et al., 2010).

Taking all this information into consideration, we sought to determine whether MiH can improve embryo quality in suboptimal culture environment.

## Materials and Methods

### Animals and Ethics

ICR mice (5–6 weeks female and 10 weeks old male) were purchased from Animal Care Facility of Nanjing Medical University and were housed in ventilated cages at constant temperature (22°C) and controlled humidity and light dark cycle. All animal experiments were approved by the Animal Care and Use Committee of Nanjing Medical University and were performed in accordance with institutional guidelines.

### Antibodies

Rabbit polyclonal anti-OCT4 antibody (Cat#: ab181557) was purchased from Abcam (Cambridge, MA, United States); mouse monoclonal anti-CDX2 antibody (Cat#:AM392-5M) was purchased from BioGenex (Fremont, United States); mouse monoclonal PAR/pADPr antibody (Cat#:4335-MC-100) was purchased from R&D Systems (Minnesota, United States). Donkey anti-Mouse Alexa Fluor 488, 555 and Donkey anti-Rabbit Alexa Fluor 555 antibodies (Cat#: A21202, A31570, A31572) were purchased from Thermo Fisher Scientific (Rockford, IL).

### *In vitro* Fertilization and Embryo Culture

To promote ovulation, female mice were intraperitoneally injected with 7 IU Pregnant Mare Serum Gonadotropin (PMSG) followed by 7 IU of Human Chorionic Gonadotropin (hCG) after PMSG priming. Cumulus-oocyte complexes (COC) were isolated from oviduct ampullae 14–15 h post-hCG injection and were cultured in drops of HTF fertilization medium under mineral oil. Sperm was collected from the tail of epididymis of adult male mice and incubated in HTF fertilization medium at 37°C in a 5% CO2 incubator for 1 h before fertilization. Then capacitated spermatozoon was added to HTF drops containing COC and co-cultured together for 6 h in the incubator to allow for fertilization. Zygotes were then washed and transferred into drops of KSOM (Aibei Biotechnology, Nanjing, M1450) medium supplemented with chemical inhibitors or not. Chemical inhibitors were used at the following concentrations: 2 μM Minocycline hydrochloride (MiH, Selleck, S4226); 20, 50 or 100 nM Olaparib (Ola, APExBio, A4154); 0.2, 0.5 or 1 nM Talazoparib (Tala, APExBio, A4153). Embryos were observed and imaged with an inverted phase-contrast microscopy (Nikon Ts2R, Japan).

### Immunostaining

Embryos were fixed in 4% paraformaldehyde for 15 min and then were permeabilized with PBS containing 0.2% Triton X-100 for 10 min at room temperature (RT). After being blocked for 1 h in blocking buffer, which comprised of PBS together with 0.1% BSA, 0.01% Tween-20 and 2.5% donkey serum, embryos were incubated with primary antibodies diluted in blocking buffer overnight at 4°C. Embryos were then washed for three times with PBS and labeled with secondary antibodies in the dark for 1 h at RT. Samples were then washed for three times with PBS and stained with 1 μg/ml DAPI for 5 min, and washed for three times before mounting on glass slides in small drops of antifade medium. Samples were then imaged using an inverted phase-contrast microscopy (Nikon Ts2R, Japan).

### *In Vitro* Exposure of Mouse Zygotes and Developmental Potential Tests Beyond Preimplantation

Zygotes were collected in drops of HTF medium after fertilization and place them on the warmed microscope stage for 1 h at 37°C. Zygotes were then transferred to the KSOM medium with or without MiH to culture.

After 100 h, the blastocysts of the above three groups were randomly selected and surgically transferred into the uteri of pseudopregnant female mice. 9 days after transferring, mice were euthanized to see whether they were pregnant.

An IVC assay was also carried out to observe the developmental potential *in vitro* by culturing the rest of blastocysts in the afore mentioned experiments according to a protocol we used before (Zhao et al., 2021). All the blastocysts were embedded in Matrigel drops and culture for further 120 h to see whether they could form egg cylinder structures.

### Mouse Zygotes Model for Oxidative Damage

Zygotes were incubated in HTF fertilization medium containing 0.1 mM H2O2 for 1 h at 37°C, then washed with fresh HTF fertilization medium three times to remove H2O2 and transferred into KSOM medium for further culture. NAC group of ROS measurement experiment were performed as control by addition of N-Acetyl-l-cysteine (Sigma-Aldrich, A9165) to a working concentration of 5 mM.

### TUNEL Assay

TUNEL assay was carried out to analyze apoptosis of embryos using One Step TUNEL Apoptosis Assay Kit (Beyotime, C1086) in accordance with the instruction manual. Embryos were fixed in 4% paraformaldehyde for 30 min and permeabilized in PBS containing 0.5% Triton X-100 for 5 min. Then embryos were incubated in the TUNEL reaction mixture (containing FITC-conjugated dUTP and terminal deoxynucleotidyl transferase) at 37°C in the dark for 1 h. The reaction was terminated by washing in washing buffer (containing 0.1% Tween-20 and 0.1% BSA in PBS) for three times. Finally, the embryos were stained with DAPI for 5 min at RT and washed before mounting on glass slides. The TUNEL labeling was observed using a fluorescence microscope (Nikon Ts2R, Japan).

### Detection of ROS Content in Embryos

2,7-dichlorodihydrofluorescein diacetate (DCFH-DA) was used to evaluate the intracellular ROS level in embryos. Embryos were incubated in KSOM medium supplemented with 10 μM DCFH-DA for 30 min at 37°C in a 5% CO2 incubator and stained with Hoechst 33342 for 10 min. Fluorescence was observed under a Laser Scanning Confocal Microscope (LSM 710, Zeiss, Germany) at a 488 nm excitation wavelength and analyzed with the Image J software.

### Statistical Analysis

Statistical analysis was performed using GraphPad Prism 7 software and all results were presented as means ± standard deviation from three independent experiments. Data were analyzed with the Student’s t-test. ∗0.01<*p* < 0.05; ∗∗*p* < 0.01; no labeling indicates no statistical significance.

## Results

### MiH-Treated Embryos Developed Slower During Cleavage Stage but Formed Blastocyst Normally

**FIGURE 1:**
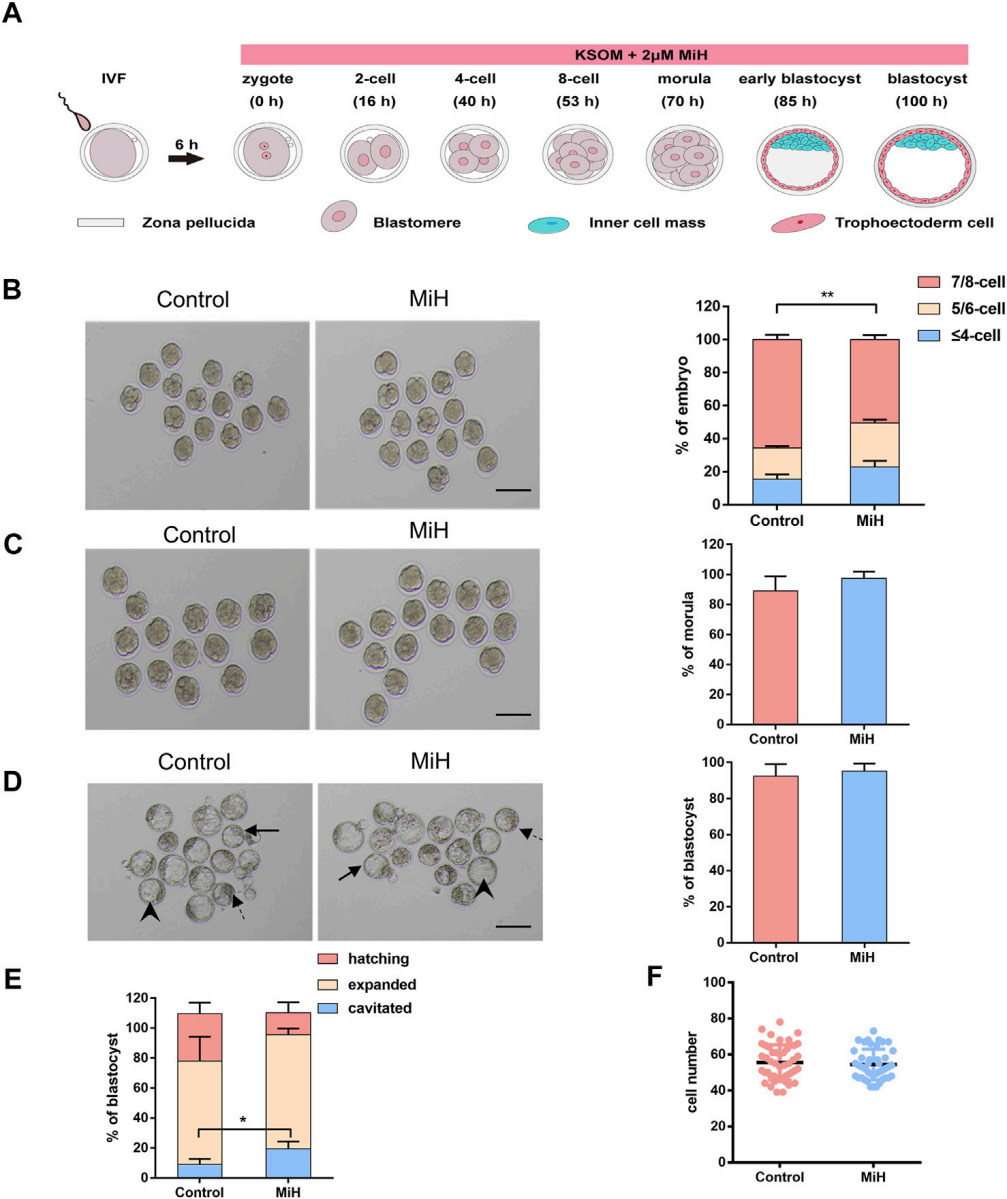
MiH-treated embryos developed slower during cleavage stage but formed blastocyst normally. **(A)** Time scheme of *in vitro* fertilization (IVF) and main procedure of MiH treatment assay of mouse embryos. **(B–D)** Representative images and frequencies of embryos observed at 53 h **(B)**, 70 h **(C)** and 100 h **(D)** after fertilization in control (*n* = 65) and MiH-treated (*n* = 60) groups. Bar = 100 μm. **(E)** Quantitative analysis of cavitied, expanded and hatching blastocysts at 100 h. Dotted arrow, arrowheads and arrows indicated the cavitied, expanded and hatching blastocysts respectively. **(F)** Cell number of each expanded or hatching blastocyst at 100 h. Data are presented as mean ± SD in three independent experiments and student’s t tests are used for statistical analysis. ∗0.01<*p* < 0.05; ∗∗*p* < 0.01; no labeling indicates no statistical significance.

Firstly, to decipher the effect of MiH on preimplantation mouse embryos, *in vitro* fertilization (IVF) was employed to obtain zygotes, which were randomized into two groups and cultured with KSOM medium in the absence (control group) or presence of 2 μM MiH (MiH group) till most of them developed into blastocysts at 100 h after fertilization (Figure 1A). We found that almost all embryos in MiH group progressed normally at 2-cell and 4-cell stages (Supplementary Figure S1A) and there were no statistical differences in the rates of 2-cell or 4-cell embryos to total zygotes at 16 h or 40 h, respectively (Supplementary Figures S1B,C).

However, MiH-treated embryos showed slower progression of development since 53 h, with only 50% of embryos being consisted of 7–8 cells while that of control group was nearly 65% (Figure 1B). Then nearly 90% of embryos in both groups would undergo compaction and continue to form morula at 70 h (Figure 1C). In order to count the cell number of each embryo, we stained nuclei with DAPI and found that the count of each morula in MiH group was significantly fewer than that in control group (Supplementary Figures S1C,D), indicating a slower developmental kinetics as well. The difference became more evident at 85 h, with only 45% of zygotes formed blastocysts, whereas the efficiency of control group was about 70% (Supplementary Figures S1A,C). Notably, the blastocyst formation efficiencies of the two groups were comparable at 100 h (Figure 1D).

In order to further determine whether there was any other difference in the blastocysts, we classified blastocysts into three types depending on their expansion level as cavitated, expanded and hatching. The cavitated blastocyst is one whose blastocele has already formed, but it later continues to fill with fluid so that the blastocyst can expand. When it is expanded, the blastocyst is larger and the zona pellucida is thinner, so hatching can begin (Figure 1D). We analyzed the frequencies of these three types of blastocysts in the two groups and found a higher ratio of cavitated blastocyst in the company with lower ratios of expanded and hatching blastocyst in MiH group without statical significance (Figures 1D,E), which could be attributed to the slower developmental rate. However, the total cell number per blastocyst was comparable between the ones in control and MiH group at 100 h (Figure 1F). Here, cavitated blastocysts in the two groups were excluded for statistics because of the great individual variation. Combined these data, we could not exclude the possibility that the above phenomenon was caused by slower pumping of fluid into the blastocyst cavity.

In brief, MiH would develop slower during cleavage stage but formed blastocyst with similar efficiency at last. These were not consistent with findings of T. Osada*et al.* and Imamura T *et al.*, who used 3-ABA, PJ-34 and 5-AIQ. The inconsistency perhaps was related with inappropriate does and different side effects among inhibitors.

### MiH Affected Embryo Development Through Inhibiting PARP1

**FIGURE 2:**
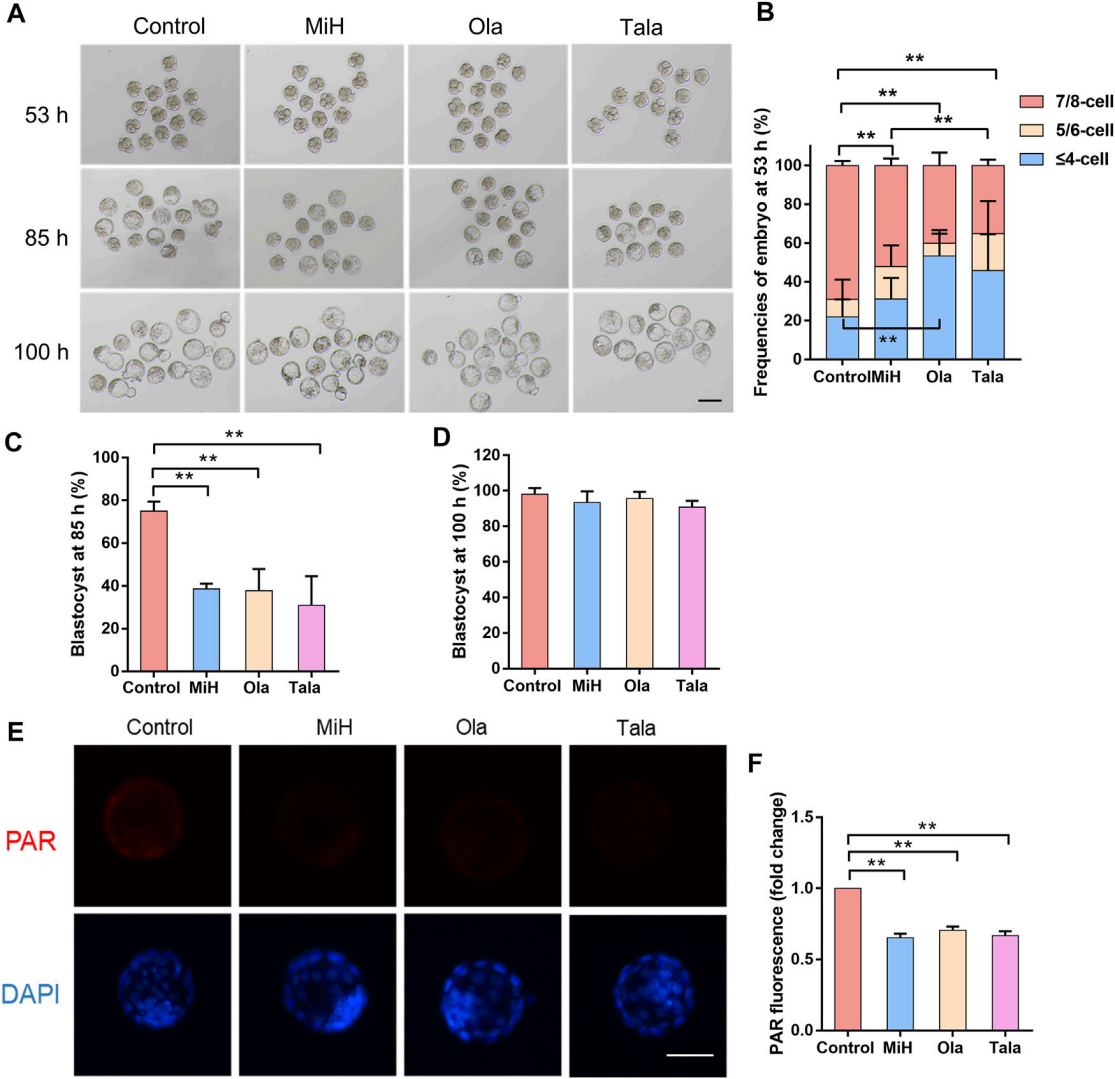
Canonical PARP1 inhibitors showed similar effects on preimplantation embryos. **(A)** Brightfield images of embryos in control, MiH-, Olaparib (Ola)- and Talazoparib (Tala)-treated groups at 53, 85 and 100 h after fertilization. Bar = 100 μm. **(B–D)** Frequencies of embryos observed at 53 h **(B)** and blastocyst formation efficiencies at 85 h **(C)** and 100 h **(D)**. *n* (Control) = 46, *n* (MiH) = 44, *n* (Ola) = 45, *n* (Tala) = 43. **(E,F)** Staining and fluorescence intensity of expanded blastocysts in the four groups for PAR. Bar = 50 μm. Data are presented as mean ± SD in three independent experiments and student’s t tests are used for statistical analysis. ∗, 0.01<*p* < 0.05; ∗∗, *p* < 0.01; no labeling indicates no statistical significance.

For the maintenance of EPS self-renewal, MiH can be replaced by other PARP1 inhibitors and *Parp1*-deficient mouse EPS could contribute to both TE and ICM in the absence of MiH (Yang et al., 2017). We further examined whether the influence of MiH on embryonic development was through inhibiting PARP1 as well. Olaparib (Ola) and Talazoparib (Tala) were two Food and Drug Administration (FDA) approved canonical PARP1 inhibitors that recommended for the treatment of various cancers (Robson et al., 2017; Litton et al., 2018). Then we treated zygotes with 20, 50 and 100 nM Ola and 0.2, 0.5 and 1 nM Tala. Nearly half of the zygotes were impaired in higher concentrations groups while embryos in 20 nM Ola and 0.2 nM Tala-treated groups progressed normally (Supplementary Figure S2). To further evaluate effects of PARP1 inhibitors at lower concentrations on embryonic development, we traced embryos in the four groups at multiple timepoints during blastocysts formation. We noticed that embryos treated with 20 nM Ola or 0.2 nM Tala showed no difference in the formation efficiencies of 2-cell nor 4-cell at 16 and 40 h respectively (Supplementary Figure S3), similar to those in MiH group (Supplementary Figure S1). However, embryos treated with PARP1 inhibitors turned to develop slower at 53 h, with only 40% of zygotes in Ola group contained 7–8 blastomeres. The ratio of Tala group was about 35%, comparable to that of MiH group but significantly less than that of control group (Figures 2A,B). The delay turned to be obvious at 85 h that the blastocyst formation rates were significantly decreased in Ola- and Tala-treated groups (Figures 2A,C), whereas the final outcome was not impaired (Figures 2A,D). Moreover, embryos treated with Tala developed most slowly among those in four groups, which might be ascribed to the most potent effects of inhibition on PARP1 (Murai et al., 2012; Murai et al., 2014).

To further address whether PARP1 was inhibited in embryos treated with MiH, Ola and Tala, PAR (poly ADP-ribose polymer), a product of PARP1 activity, was detected with an anti-PAR antibody to assess inhibition. It was previously reported that distribution of PAR remained diffused and cytosolic during preimplantation development (Imamura et al., 2004). As shown in Figures 2E,F, the distribution of PAR was diffuse and cytosolic in some cells of blastocysts in control group. On the contrary, embryos cultured in KSOM supplemented with MiH, Ola and Tala showed no signal. These results suggest that PARP1 was inhibited by MiH and two other well-known PARP1 inhibitors as previous reports.

MiH is a critical chemical compound for EPS cells maintenance (Yang et al., 2017). We hypothesized that MiH played similar roles in first cell fate decision that generated populations of outside cells and inside cells, respectively. This is a critical developmental stage because the embryo must allocate its blastomeres into either TE or ICM. To determine whether supplementation of MiH, Ola or Tala would affect the specification of TE and ICM, immunostaining for CDX2 and OCT4 was applied (Palmieri et al., 1994; Niwa et al., 2005; Strumpf et al., 2005). As shown in Supplementary Figure S4A, OCT4 was almost expressed in each blastomere, while CDX2 was only expressed in the cells which lately develop into TE at 70 h. All of the PARP1 inhibitors-treated embryos contained much more CDX2+/OCT4+ cells than untreated ones but significantly fewer CDX2-/OCT4+ cells. When blastocyst forms, OCT4 and CDX2 is restricted to ICM and TE cells separately, with several CDX2+/OCT4+ cells exist. In PARP1 inhibitors-treated blastocysts, more CDX2+ cells emerged with fewer CDX2-/OCT4+ cells remaining (Supplementary Figure S4B), indicating that PARP1 inhibition might promote specification of TE identity, similar to the effect of MiH on EPS cells.

Thus, we supposed that MiH affected embryo development through inhibiting PARP1 and boosted a trophoblast bias.

### MiH Protected Zygotes From Long-Term Exposure to External Environment

**FIGURE 3:**
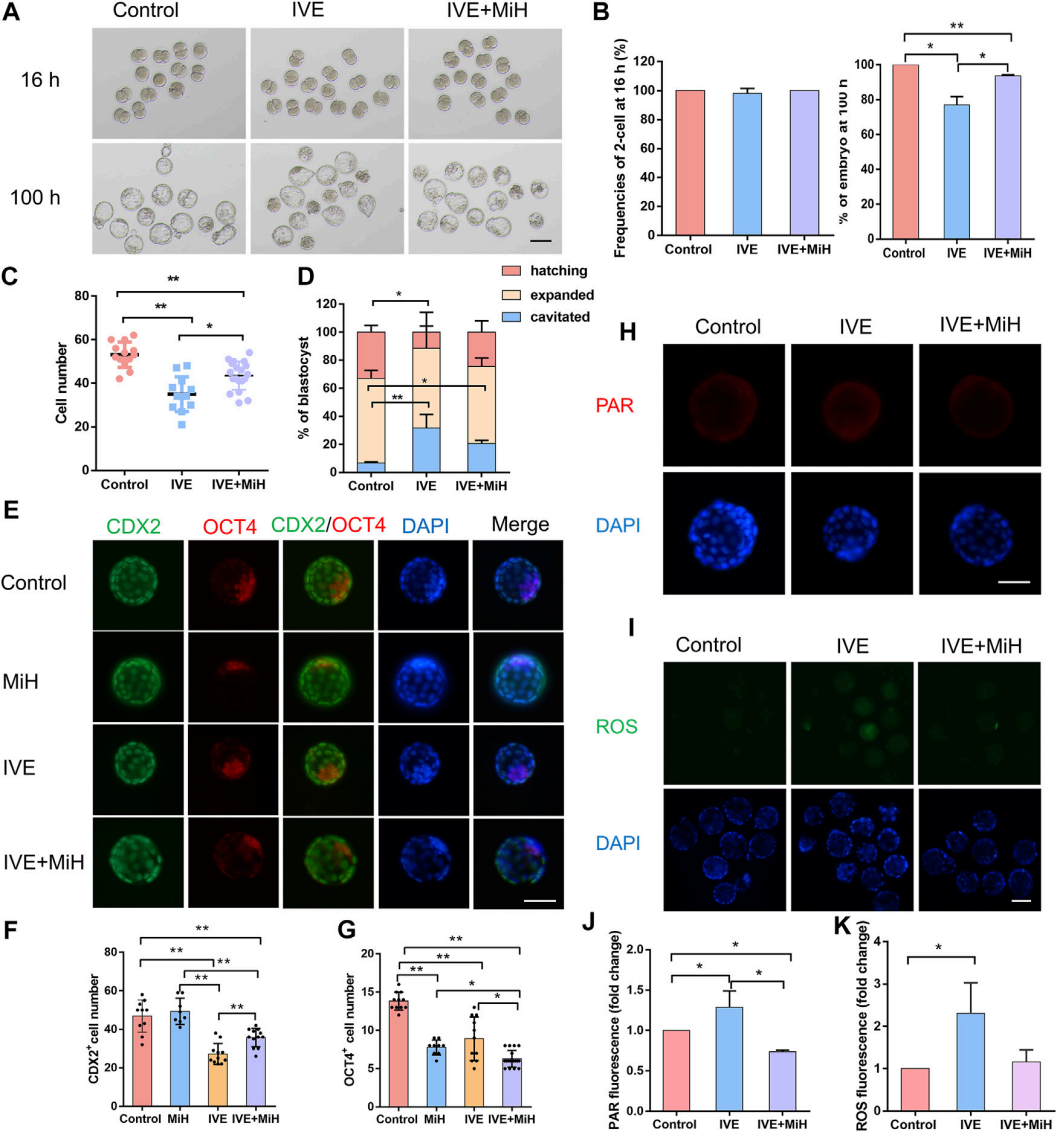
PARP1 inhibitors could protect preimplantation mouse embryos from exposure to external environment. **(A,B)** Representative images and frequencies of 2-cell embryo observed at 16 h and blastocyst at 100 h after fertilization. Control, zygotes were transferred into incubators soon after washing and cultured in KSOM condition till 100 h; IVE and IVE+MiH, zygotes were exposed to external environment for 1 h and then were transferred into incubator and cultured in KSOM without (IVE) or with MiH supplemented (IVE+MiH). Bar = 100 μm. **(C)** Comparison of cell numbers of blastocyst at 100 h. **(D)** Ratios of cavitated, expanded and hatching blastocysts at 100 h. 46, 49, 47 embryos were totally counted in Control, IVE and IVE+MiH groups. **(E–G)** Immunofluorescent images of blastocysts and cell numbers in each blastocyst at 100 h. CDX2 (green) and OCT4 (red) were used as markers for TE and ICM separately. Bar = 50 μm. *N* (Control) = 12, *n* (IVE) = 11, *n* (IVE+MiH) = 18. **(H)** Staining images of expanded blastocysts in the three groups for PAR. Bar = 50 μm. **(I)** Fluorescent images of ROS for expanded blastocysts. Bar = 100 μm. **(J,K)** Quantification intensity levels for PAR **(J)** and ROS **(K)** in in Control, IVE and IVE+MiH groups. Data are presented as mean ± SD in three independent experiments and student’s t tests are used for statistical analysis. ∗0.01<*p* < 0.05; ∗∗*p* < 0.01; no labeling indicates no statistical significance.

*In vitro* fertilization (IVF) is an effective clinical strategy for the couple who fail to conceive. While the percentage of successes is much higher nowadays, one of the most plausible causes of the failure of IVF procedures is the poor quality of gametes leading to aberrant embryonic development. Previous Studies have shown that psychological stress can exert detrimental effects on reproduction in women. *In vitro* fertilization techniques, in particular, gametes collection, manipulation, and culture may generate stress environment which would cause oxidative stress. Furthermore, some studies suggested that inhibited PARP activity could protect against the loss of cell viability, preserve NAD + levels and improve cellular bioenergetics in *in vitro* experiments in U937 cells subjected to oxidative stress (Ahmad et al., 2019). Thus, we wonder whether MiH could restore the outcome of the embryos under stressful environment. To verify this hypothesis, we placed zygotes on the warmed microscope stage for 1 h, then cultured them in medium in the absence (*in vitro* exposure, IVE) or presence of MiH (IVE+MiH). The ratios of 2-cell and 4-cell embryos were strikingly resembling in all groups (Figures 3A,B, Supplementary Figures S5A,B). Since 53 h, zygotes in IVE and IVE+MiH groups developed slowly, with no more than 50% of them reaching 8-cell stage while the ratio of control group was 80% (Supplementary Figures S5A,C). Later, nearly 90% of zygotes in IVE+MiH group developed into morula, which was much higher than that of IVE group and was comparable to control group (91 vs. 96% control; 83 vs. 96% control) (Supplementary Figures S5A,D). Though long-term exposure to external environment significantly reduced blastocyst formation efficiencies of embryos in both IVE and IVE+MiH groups at 85 h (Supplementary Figures S5A,E), the supplement of MiH rescued the final blastocyst formation potential of embryos in IVE+MiH group, with equivalent ratio (93%) to that of control group, but significantly higher than that of IVE group (77%) at 100 h (Figures 3A,B). Additionally, the average cell number of blastocysts in IVE group was markedly less than that in control and IVE+MiH groups (Figure 3C). Analogously, the frequency of hatching blastocyst in IVE group decreased dramatically than that of control and IVE+MiH groups, while the ratio of cavitated blastocyst exhibited much higher in IVE group (Figure 3D). All these data above suggested that MiH played a protective role for preimplantation embryos in the process of suffering long-term exposure to environment.

In order to further address whether these recovered blastocysts possessed normal developmental potential beyond preimplantation stage, they were randomly selected from the three groups and surgically transferred into the uteri of pseudopregnant female mice. 9 days after transferring, only one which had been transferred with blastocysts in IVE+MiH group was pregnant, indicating that MiH may partly rescue the embryo developmental potential from long time exposure to the external environment (Supplementary Figure S6). However, we could not make a very solid conclusion that embryos in this group possessed better developmental potential beyond preimplantation stage, due to the limited sample size and failure of control group. Actually, a satisfyingly pregnant rate of embryo transplantation experiment will be inevitably affected by the uncertainty of embryo implantation even in the presence of what appears to be a receptive endometrium. It is well known that late blastocyst development is delayed in embryos in *in vitro* culture relative to their *in vivo* counterparts. However, we had to culture all of the embryos for 100 h to ensure the restoration of the suboptimal environmental exposure, perhaps resulting in the miss of appropriate transplanting time, especially for those unexposed ones. This perhaps in turn led to embryo-endometrial desynchronization of control group.

We also performed an IVC assay to observe the developmental potential *in vitro* by culturing the rest of blastocysts in the afore mentioned experiments according to a protocol we used before (Zhao et al., 2021). However, the egg cylinder formation efficiencies of all the three groups were as low as less than 10%, hardly to say whether IVE+MiH blastocysts obtained better further developmental potential. When the natural fertilized and *in vivo* developed E3.5 embryos were used, in the previous studies, the efficiencies were only 30–40% as well (Bedzhov et al., 2014; Zhao et al., 2021), which could partly explain the low efficiency in our experiments. Though both the transplantation and IVC assays were statistically not conclusive, they provide some hints of what to expect if the experiments were performed many times.

Clinical data collected in recent years proved that embryo culture impaired embryonic developmental potential including delaying cell cycle kinetics and reducing TE cells in blastocysts (Giritharan et al., 2007). We then asked whether IVE affected the specification of TE and ICM and observed significant reduction of CDX2 positive cell numbers of blastocysts in both IVE and IVE+MiH groups comparing with those in control and MiH groups. However, blastocysts in IVE+MiH group contained much more CDX2 positive cells than those in IVE group, implying that MiH might protect TE cells from environmental stress (Figure 3F). Meanwhile, blastocysts in all three groups had much less OCT4 positive cells when they were compared with ones in control group, with great variations between individuals in IVE group (Figure 3G). Hence, we supposed the protective effect of MiH might be partly attributed to its boost of cells into a TE fate.

Long term exposure to external environment may lead to unexpected pH and temperature shifts in the embryo culture medium. Alternation in pH has been shown to influence intracellular homeostasis, with particular effects, including protein synthesis, mitochondrial function, cellular metabolism, and cytoskeletal remodeling (Will et al., 2011). Temperature is another important factor for embryonic development, which will influence integrity of spindles and DNA fragmentation. It has been reported that over-activation of PARP1 led to apoptotic and necrotic cell death during Myocardial ischemia-reperfusion injury (Raedschelders et al., 2012). To confirm whether long term exposure to external environment would cause PARP1 over-activation, we performed immunodetection of PAR and found a significant stronger signal in embryos of IVE group, which exhibited weaker in embryos of both control and IVE+MiH groups (Figures 3H,J).

Besides, unavoidable environmental factors, such as light exposure, excess temperature and pH fluctuation of culture medium, which increase ROS production, have been recognized to negatively affect embryo developmental potential and result in suboptimal pregnancy rates (Agarwal et al., 2005a). For this reason, we wondered whether long term exposure to external environment induced the production of ROS. To verify this problem, we explored the intracellular ROS level at 100 h. Fluorescent analysis showed robust signal in the embryos of IVE group while supplementing culture medium with MiH could restore it to normal level (Figures 3I,K). Thus, MiH might reduce the adverse effects of long-term exposure to external environment on embryos through inhibiting the generation of ROS.

### PARP1 Inhibitors Might Improve Embryo Viability Against Oxidative Injury

**FIGURE 4:**
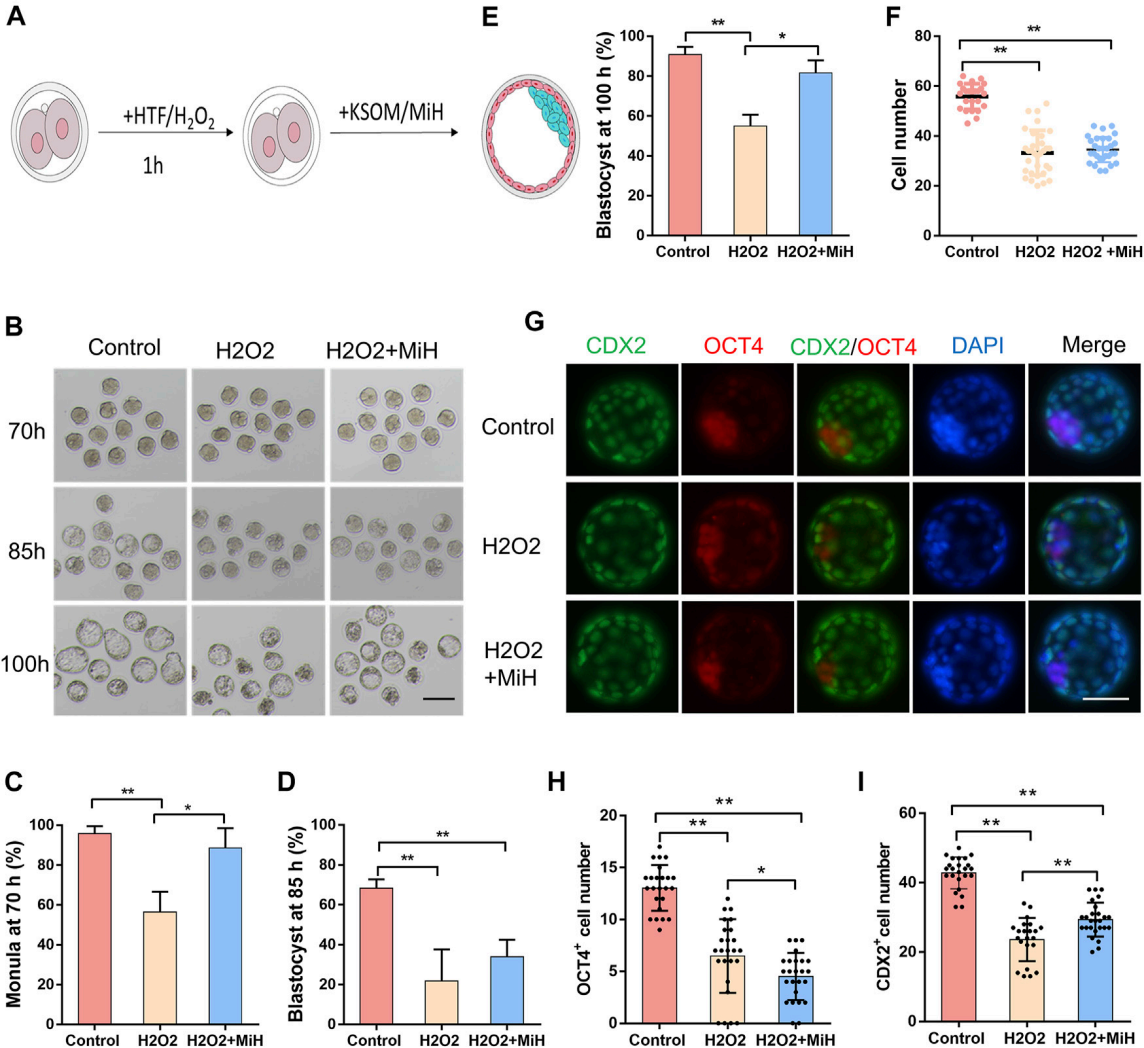
MiH improved mouse preimplantation embryos viability against oxidative stress. **(A)** Main procedure of oxidative embryo model and MiH treatment assay. **(B)** Representative images of embryos in control and H2O2-treated groups with or without MiH at 70, 85, 100 h after fertilization. Bar = 100 μm. Control referred to embryos who had not suffered from H2O2 treatment and cultured under KSOM; H2O2 and H2O2+MiH referred to zygotes who were treated with 0.1 mM H2O2 for 1 h and then cultured under KSOM in the absence (H2O2) or presence of MiH (H2O2+MiH), respectively. **(C–E)** Morula or blastocyst formation efficiencies at 70 h **(C)**, 85 h **(D)** and 100 h **(E)** after fertilization. 44, 47, 45 embryos were totally counted in control, H2O2- and H2O2+MiH-treated groups, respectively. **(F)** Comparison of cell numbers of blastocyst at 100 h **(G–I)** Immunofluorescent images of blastocysts at 100 h. CDX2 (green) and OCT4 (red) were used as markers for TE and ICM separately. Bar = 50 μm. *N* (Control) = 26, *n* (H2O2) = 34, *n* (H2O2+MiH) = 31. Data are presented as mean ± SD in three independent experiments and student’s t tests are used for statistical analysis. ∗0.01<*p* < 0.05; ∗∗*p* < 0.01; no labeling indicates no statistical significance.

Having shown that MiH could partly reduce ROS level in embryos that exposed to external environment, we assessed whether PARP1 inhibitors would improve embryo viability against oxidative damage. Hydrogen peroxide (H2O2) is one of the strongest oxidants and will lead to overproduction of ROS. However, increased ROS production induces multiple cellular damages and mitochondrial alternation, which consequently disturbs embryonic development of preimplantation embryos *in vitro* (Liu et al., 2000; Kitagawa et al., 2004; Zhang et al., 2010). Accordingly, zygotes were randomly divided into untreated and treated groups. In the treated group, zygotes were exposed to 100 μM H2O2 for 1 h, washed extensively, and then cultured in medium with (H2O2+MiH) or without MiH (H2O2) while untreated zygotes were incubated in HTF for 1 h and then cultured in KSOM medium (Control) (Figure 4A). Since 40 h, H2O2-treated zygotes in both two groups (H2O2+MiH and H2O2) exhibited lower rates of 4-cell and 8-cell stages embryos (Supplementary Figure S7). Only zygotes in H2O2 group showed decreased rates of morula at 70 h (Figures 4B,C). Furthermore, though blastocyst formation efficiencies of H2O2+MiH, H2O2-treated zygotes were both much lower than those of non-treated ones at 85 h (Figure 4D), rate of H2O2+MiH group was restored to the equivalent level of control group at 100 h (Figure 4E). Morphologic analysis also revealed that treatment of zygotes with H2O2 induced fragmentation and developmental retardation during this process, while embryos in H2O2+MiH and control group exhibited less (Figure 4B). Moreover, the average cell numbers of blastocysts at 100 h significantly decreased in the H2O2 and H2O2+MiH groups than that of control group (Figure 4F). To further address the influence of H2O2 on cell fate decision, embryos were immunostained with CDX2 and OCT4 antibodies. We found that though CDX2 positive cell numbers decreased both in H2O2 and H2O2+MiH treated ones, blastocysts in H2O2+MiH group had much more CDX2 positive cells than those in H2O2 group. By contrast, no restoration of OCT4 positive cells were found in H2O2+MiH treated ones (Figures 4G–I), implying that the protective effect of MiH was associated with protection for TE cells.

**FIGURE 5:**
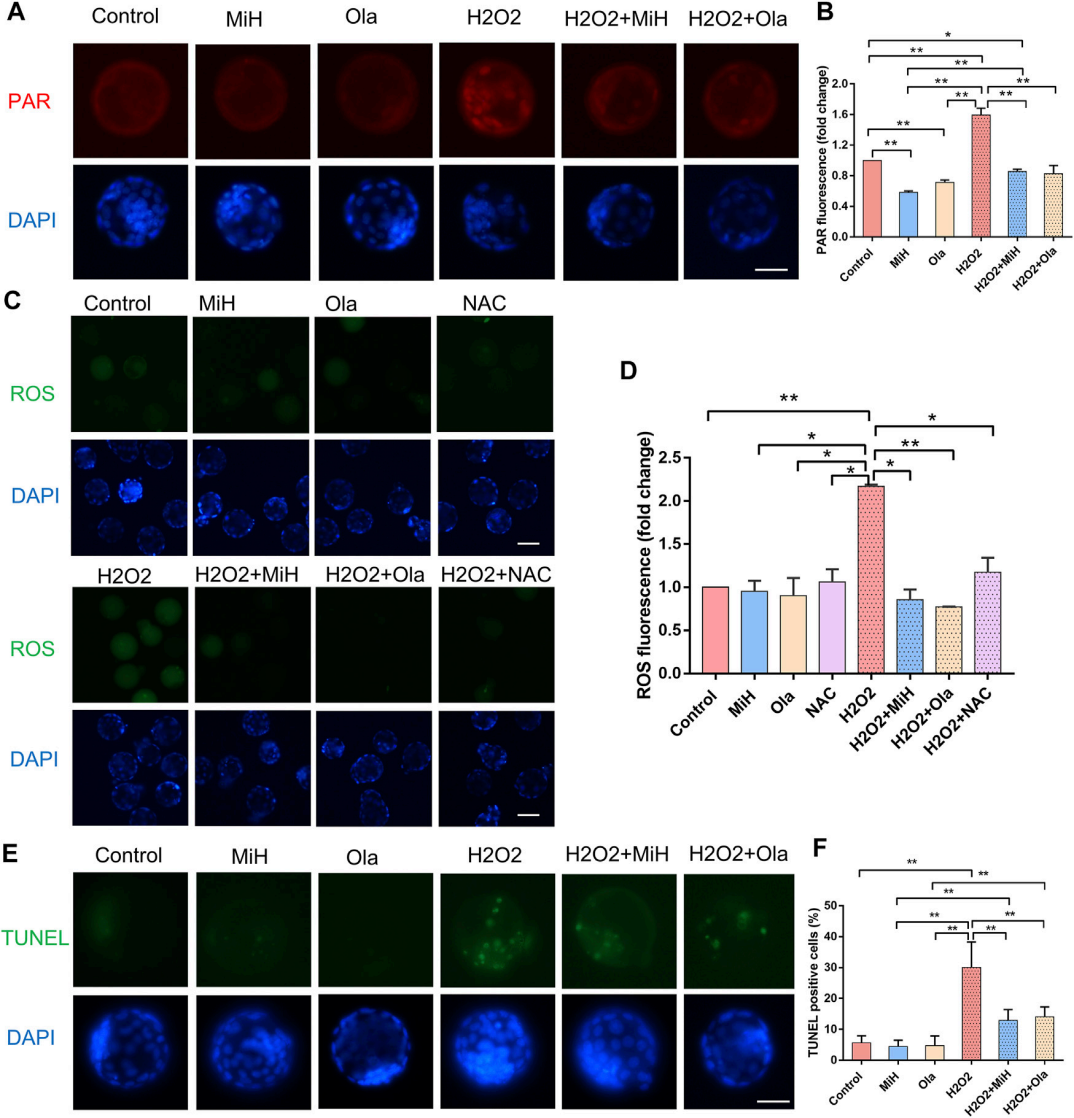
PARP1 inhibitors could protect mouse embryos from ROS and reduced cell apoptosis. **(A,B)** Staining and intensity levels for PAR of expanded blastocysts in untreated and H2O2-treated groups supplemented MiH, Ola or not. **(C,D)** Fluorescent images and intensity levels of ROS in expanded blastocysts. **(E,F)** Representative fluorescent images of apoptotic cells **(E)** and quantitative analysis of TUNEL-positive blastocysts **(F)**. Data are presented as mean ± SD in three independent experiments and student’s t tests are used for statistical analysis. ∗0.01<*p* < 0.05; ∗∗*p* < 0.01; no labeling indicates no statistical significance. Bars = 50 μm.

Additionally, to confirm whether the antioxidative effect of MiH was through inhibition of PARP1, we firstly explored the level of PAR in the expanded blastocysts. As shown in Figures 5A,B, we found that H2O2 exposure induced nucleic localization signal of PAR. But in H2O2+MiH and H2O2+Ola groups, almost no signal was found in blastocysts, which was consistent with that in all non-treated groups. Combined with the data that no PAR signal emerged in H2O2-untreated groups, we indicated that MiH and Ola would inhibit PARP1 overactivation which was induced by H2O2 treatment. Then, we used N-acetyle-cysteine (NAC), a conventional antioxidant for ROS inhibition, as a positive control to investigate whether MiH could decrease ROS level in H2O2-treated embryos like NAC did. These results showed that H2O2 treatment increased ROS signal in embryos, but both PARP1 inhibitors and NAC supplementation could recover the ROS level (Figures 5C,D).

A TUNEL assay was also performed to establish whether MiH reduced apoptosis rate in blastocysts developed from H2O2-treated zygotes. As expected, H2O2 caused severe apoptosis in embryos in comparison with those in non-treated ones, however, blastocysts in H2O2+MiH and H2O2+Ola groups possessed much fewer apoptotic cells than those in H2O2 group, suggesting a protective role of MiH against apoptosis in embryonic development (Figures 5E,F).

## Discussion

It is well-established that though lots of technical advances have been achieved in the administration of *in vitro* fertilization (IVF) to infertile couples, embryo quality is still a major contributing factor to the outcomes of IVF cycles (Moragianni et al., 2019). During IVF, the development of human preimplantation embryos progresses in an artificial environment and is tightly controlled by extrinsic factors during inevitable processes of handling, manipulation and culture of gametes and embryos. Clinical data collected in recent years suggested that embryo culture impaired embryonic developmental potential including delaying cell cycle kinetics and reducing trophectoderm cells (TE) in blastocysts (Giritharan et al., 2007). As the trophoblastic cells develop into placenta, decrease of trophectoderm cell number may partly explain why *in vitro* cultured animals’ embryos exhibit impaired placental development and function, and thus fetal growth (Bloise et al., 2012; Chen et al., 2015; Tan et al., 2016). Chen et al. (2019) also showed that mouse blastocysts which were cultured *in vitro* after fertilization had fewer numbers of total cells, TE cells and ICM cells than those developed *in vivo*. In order to mimic the evitable and frequent removal from the incubator for assessment in clinical IVF laboratories, we placed zygotes on a hot-stage microscope for 1 h and cultured them in traditional KSOM embryonic culture medium in incubator (IVE) till 100 h. This would induce a lot of external factors such as light, temperature, reduced oxygen tension and pH fluctuation of culture medium that contributed to the outcome of embryonic development. Consistently with these aforementioned reports, no more than 80% of IVE embryos formed blastocyst and none implanted in the transplantation assay (Supplementary Figure S6), with reduction in total cells, TE cells and ICM cells as well (Supplementary Figures S3A–C, Supplementary Figures S3E–G, Supplementary Figures S5C–E). Though average OCT4 positive cell numbers of IVE embryos did not decrease when being compared with those of MiH-treated ones, they varied a lot among each blastocyst (Figure 3G). In this procedure, the accumulation of ROS was trigged (Figures 3I,K) in IVE embryos, suggested that this long-term exposure to external environment might impair embryonic developmental potential through oxidative stress.

Among numerous external factors contributing to embryonic development, O2 concentration is important to human embryonic development (Gardner 2008). Previous studies have shown that oxygen tension is found to range from 2 to 8% in the oviduct and uterus of most mammalian species (Yedwab et al., 1976; Fischer and Bavister 1993). In addition, numerous studies suggest that embryo development can be improved by culturing embryos under low O2 tension. However, only around 25% of IVF cycles worldwide are performed exclusively under 5% oxygen, with 34% of clinics reporting using 5% oxygen for specific embryonic culture stages. A significant percentage of clinical laboratories are still using atmospheric oxygen concentrations (20%) for the culture of human embryos (Christianson et al., 2014). Besides, unavoidable environmental factors which elevate ROS level are recognized to impair embryonic developmental potential and result in suboptimal pregnancy rates (Agarwal et al., 2005a). In this study, zygotes were exposed to H2O2, which would result in overproduction of ROS (Figure 4A). In agreement with multiple reports, zygotes in H2O2 group exhibited impaired developmental potential, including developmental delay (Figures 4B,C and Supplementary Figure S7), blastocyst formation efficiency (Figures 4D,E) and cell number decrease (Figure 4F), whereas the number of apoptotic cells increased (Figure 5E). Meanwhile, an intracellular accumulation of ROS and PAR was observed (Figures 5A–D), reminding a relationship between oxidative stress and PAR.

Indeed, in response to high glucose exposure *in vitro* or diabetes and hyperglycemia *in vivo*, ROS generation occurs and promotes the formation of large amount of DNA single-strand breakages which trigger rapid over-activation of PARP1 and lead to inflammation, apoptotic and necrotic cell death (Ansley D M et al., 2012). PARP1 in turn depletes the intracellular concentration of its substrate, NAD+, slowing the rate of glycolysis, electron transport, and ATP formation. In addition to the direct cytotoxic pathway regulated by DNA injury and PARP1 activation, PARP1 also appears to modulate the course of inflammation by regulating the activation of NF-κB (Du et al., 2003). PARP1 overexpression was also shown to be involved in heart failure (Xiao et al., 2005).It had been reported that heart dysfunction was associated with an increase in poly (ADP-ribosyl)ation in mouse and rat models of diabetes (Pacher et al., 2002). Genetic deletion or pharmacological PARP1 inhibition was shown to protect diabetic heart and ameliorates metabolic dysfunction (Pacher et al., 2002; Qin et al., 2016; Zakaria et al., 2016). For example, INO1001, a highly potent PARP1 inhibitor, could prevent oxidative stress and improve nephropathy in diabetic mice (Szabo et al., 2006) and relieve aging-associated cardiac and vascular dysfunction (Radovits et al., 2007). In current study, H2O2 treatment boosted super activation of PARP1 with robust accumulation of ROS while that in PARP1 inhibitors-treated embryos (MiH+H2O2) recovered to normal level (Figure 5D). Moreover, blastocyst formation efficiencies of H2O2-treated zygotes were all much lower than those of non-treated groups (Figures 4B,D) at 85 h, while that of H2O2+MiH group recovered to the similar level of control group at 100 h (Figures 4B,E). These were in line with the aforementioned reports (Szabo et al., 2006; Radovits et al., 2007), indicating that PARP1 inhibitors could protect preimplantation embryos from oxidative stress through preventing PARP1 overexpression. Studies have shown that high level of ROS has adverse effects on the quality of oocyte and embryo growth. Besides, embryos are highly sensitive to environmental variables or oxidant levels. As mentioned in a number of reports, under different stress conditions, massive DNA damage can lead to excessive activation of PARP1 (Rodriguez-Vargas et al., 2012; Cieslik et al., 2013), which has been previously proposed being crucial to neuronal death through mechanisms linked to NAD depletion and release of apoptosis inducing factor from the mitochondria (Alano et al., 2006). PARP1 activity rapidly increases, thus leading to the formation of long-chain poly (ADP-ribose) (PAR) (Schreiber et al., 2006). In our experiments, we found that both MiH and Ola could significantly reduce the fluorescence intensity of PAR in H2O2 treated blastocysts (Figures 5A,B). Furthermore, TUNEL results showed that MiH, as well as Ola, could reduce apoptosis of blastocysts after H2O2 exposure (Figure 5E). All the results suggested PARP1 inhibitors, including MiH and Ola, which might act as an antioxidant property, attenuates oxidative damage by directly inhibiting PARP1 activity. These results suggest a mechanism by which MiH might improve efficiency of IVF techniques.

Several works have proven that supplementation of various antioxidants in culture medium can release ROS accumulation. Enzymatic and synthetic antioxidants are the main defense factors against ROS (Sikka et al., 1995). The former includes catalase (CAT), glutathione peroxidase (GPx), glutathione reductase (GSR), superoxide dismutase (SOD) and peroxiredoxins, while the latter, which is also known as natural dietary supplements and widely distributed in food, consisting of vitamins and minerals (Agarwal et al., 2005b; Xu et al., 2017; Lu et al., 2018). Although numerous studies have reported the effects of individual antioxidants on embryo development (Fujitani et al., 1997; Ali et al., 2003; Kitagawa et al., 2004; Choe et al., 2010; Silva et al., 2015), it seems that none of the available ones can fully mimic the physiological conditions of the female tract (Aviles et al., 2010). This study was the first to propose PARP1 inhibitors as novel anti-oxidative supplementations for culture of preimplantation embryos.

In mouse embryos cultured under normal physical condition, PARP1 is transiently upregulated by fertilization. Though decreases at late 1-cell stage, it maintains until blastocyst stage. Meanwhile, PAR polymer is present in all stages of pre-implantation development (Imamura et al., 2004). Previous reports have shown that PJ-34 and 5-AIQ, two PARP inhibitors, could block first cell cycle of mouse embryos while the dose were as high as 30 uM and 20 uM separately (Osada et al., 2010). However, these were inconsistent with a study using 3-ABA, another commonly used enzymatic inhibitor, in which 5 mM 3-ABA accelerated pronuclear formation but arrested embryonic development before compaction, meanwhile only 64% of untreated ones reached blastocyst stage (Imamura et al., 2004). In current study, all of the three inhibitors we used here were much specific against PARP1 and did not impair the final blastocyst formation potential (Figure 1D, Figure 2D), which was inconsistent with former reports. This perhaps could be attributed to inappropriate does and different side effects among inhibitors. The data that mice carrying a double *Parp-1/Parp-2* mutation die at the onset of gastrulation (Menissier de Murcia et al., 2003) supports that specific inhibitor of PARP1 would not disrupt development of mouse preimplantation embryos.

Collectively, the supplementation of low concentrations of PARP1 inhibitors plays dual roles in mouse embryonic development process. When embryos were cultured in normal physical condition, they only slowed down developmental kinetics of embryos during cleavage stage without disturbing their final ending. Noteworthy, PARP1 inhibitors would improve mouse zygotes developmental potential against suboptimal environment, paving the way for more in-depth studies on deciphering the multiple molecular mechanisms behind embryonic development that could be useful in assisted reproductive technology.

## Data Availability

The original contributions presented in the study are included in the article/Supplementary Material, further inquiries can be directed to the corresponding author.
