# Supplementary material for: Protective Effect of Minocycline Hydrochloride on the Mouse Embryonic Development Against Suboptimal Environment
Source: Front Cell Dev Biol. 2022 Feb 1;10:799042. doi: 10.3389/fcell.2022.799042 (PMC8844553; doi:10.3389/fcell.2022.799042)
Supplement: Supplementary file 1 [file DataSheet1.docx]

***Supplementary Material***

**1. Supplementary Figures**

**
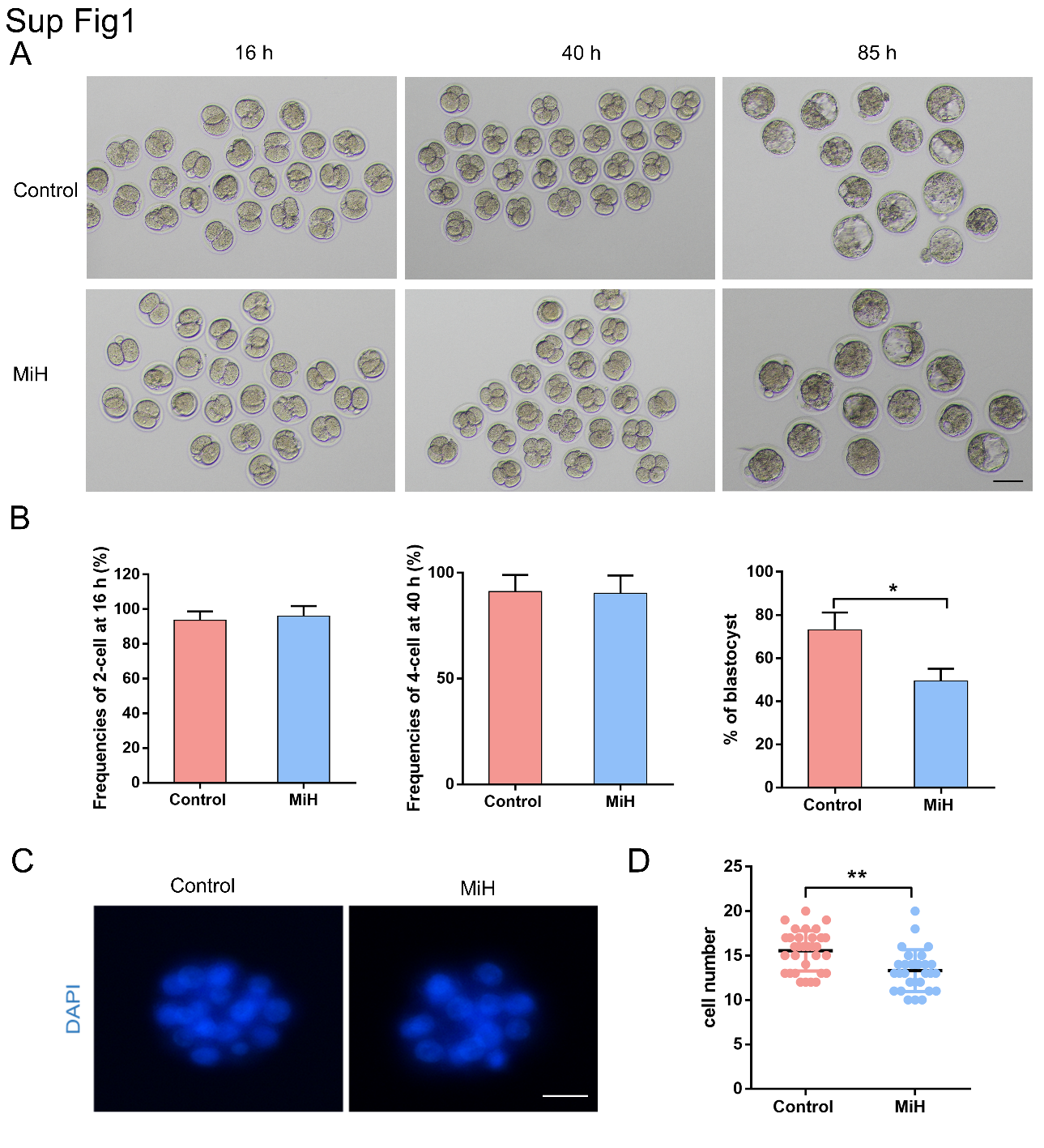
**

**Supplementary Figure 1. Observation of embryos in control and MiH-treated groups at 16, 40 h, 70 and 85 h after fertilization.** (A) Representative brightfield images of embryos at 16 h, 40 h and 85 h after fertilization. Bar=100 μm. (B) Formation efficiencies of 2-cell ,4-cell and blastocyst embryos at 16, 40 and 85 h separately. 65 embryos in control group and 60 embryos in the MiH group were counted totally. (C-D) Fluorescent images and cell numbers of morula embryos at 70 h. Bar=50 μm. Data are presented as mean±SD in three independent experiments. Student’s t tests were used for statistical analysis. No labeling indicates no statistical significance.


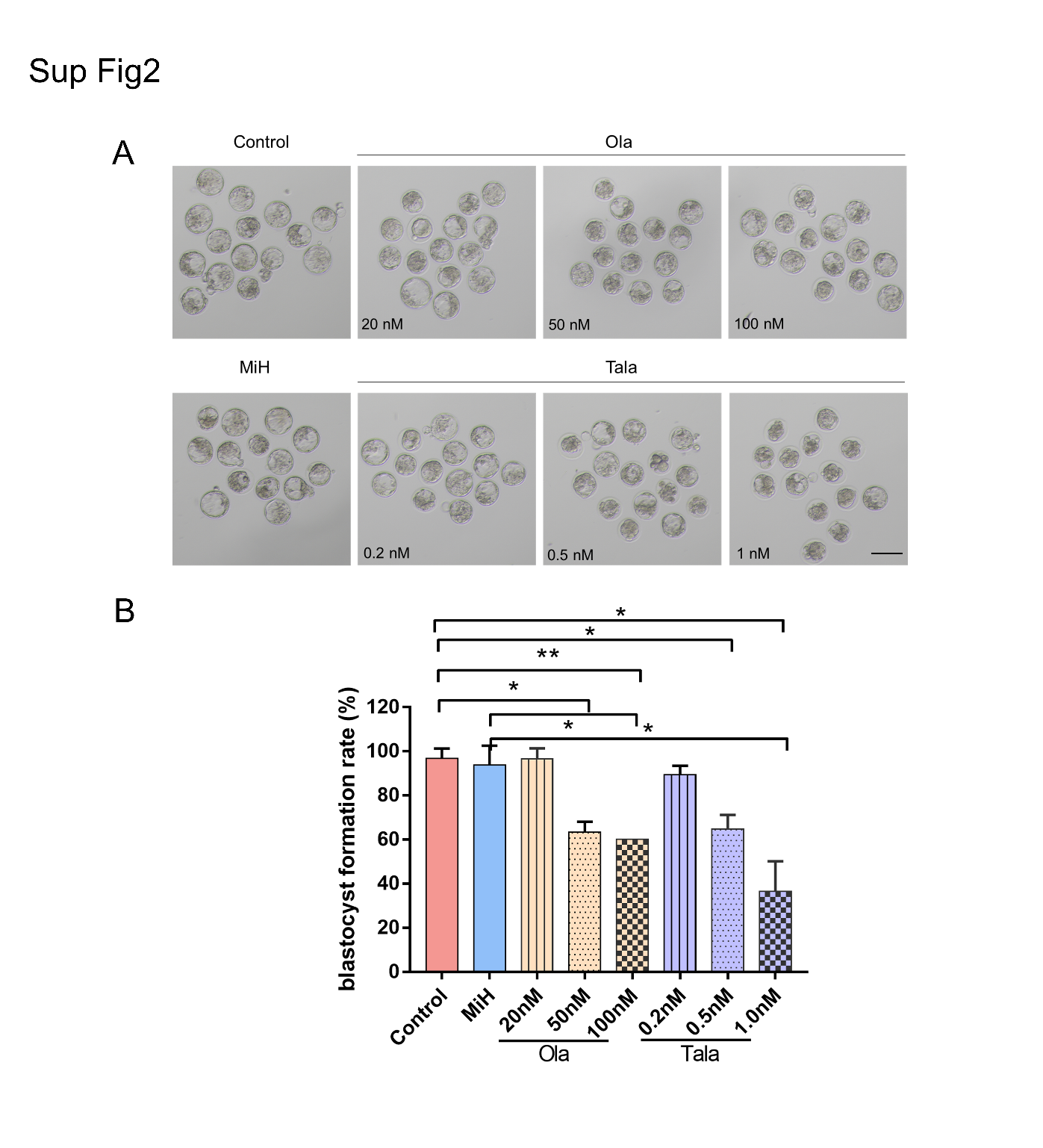


**Supplementary Figure 2. High concentrations of canonical PARP1 inhibitors reduced blastocyst formation efficiencies.** (A) Representative images of embryos at 100 h after fertilization. Bar=100 μm. (B) Blastocyst formation rates at 100 h. Control (n=31), MiH (n=30), Ola (20 nM, n=30; 50 nM, n=30; 100 nM, n=30), Tala (0.2 nM, n=28 0.5 nM, n=28; 1.0 nM, n=28). Data are presented as mean±SD in three independent experiments. Student’s t tests were used for statistical analysis. ∗, 0.01<P <0.05; ∗∗, P<0.01; no labeling indicates no statistical significance.


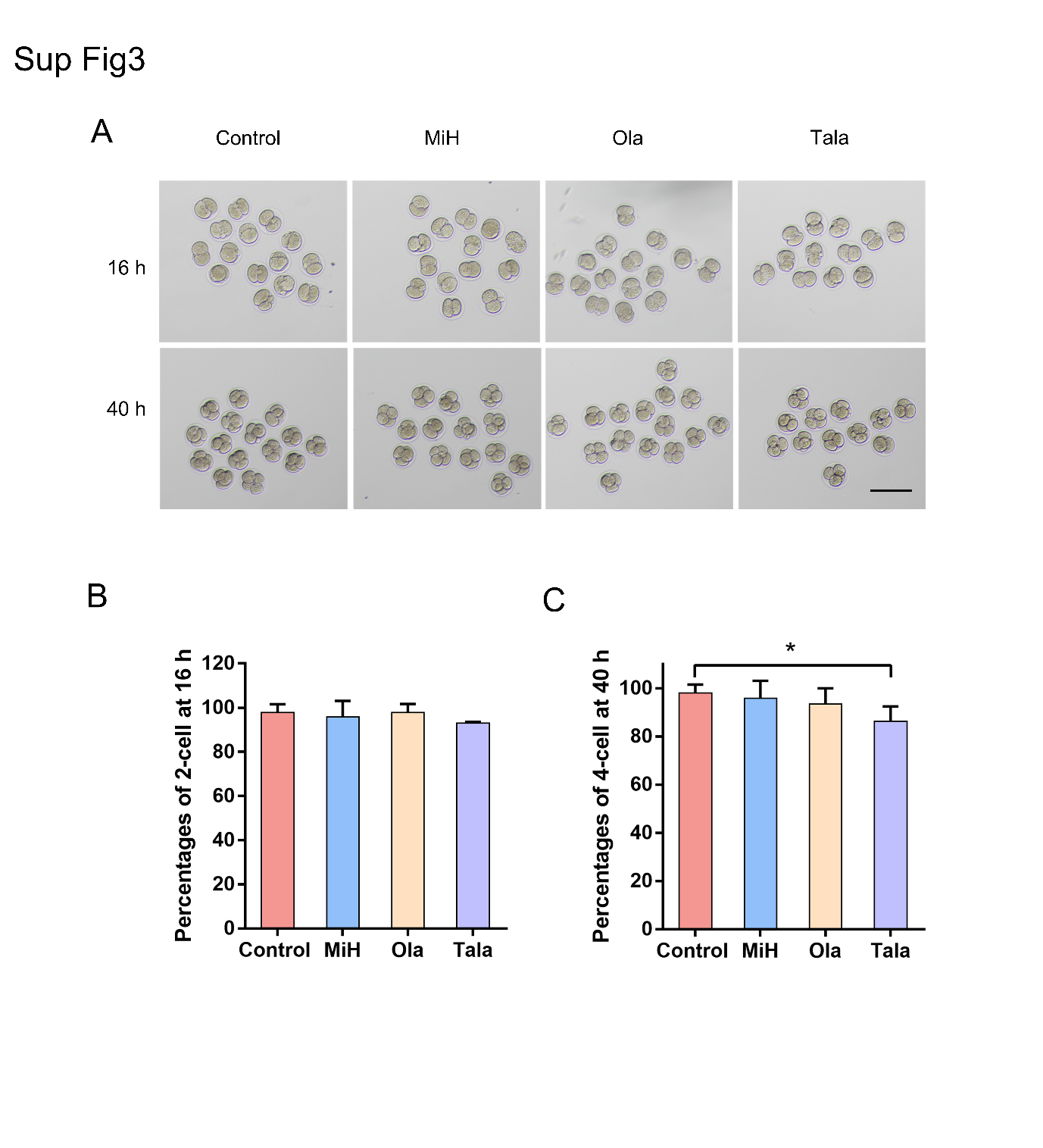


**Supplementary Figure 3. None obvious difference was found in the embryos in control, MiH- and canonical PARP1 inhibitors-treated groups.** (A) Representative images of embryos at 16 h and 40 h after fertilization. Bar=100 μm. (B-C) Formation efficiencies of 2-cell (B) or 4-cell (C) embryos separately. 46, 44, 45, 43 embryos were totally counted in Control, MiH-, Ola- and Tala-treated groups, respectively. Data are presented as mean±SD in three independent experiments. Student’s t tests were used for statistical analysis. ∗, 0.01<P <0.05; no labeling indicates no significant difference.


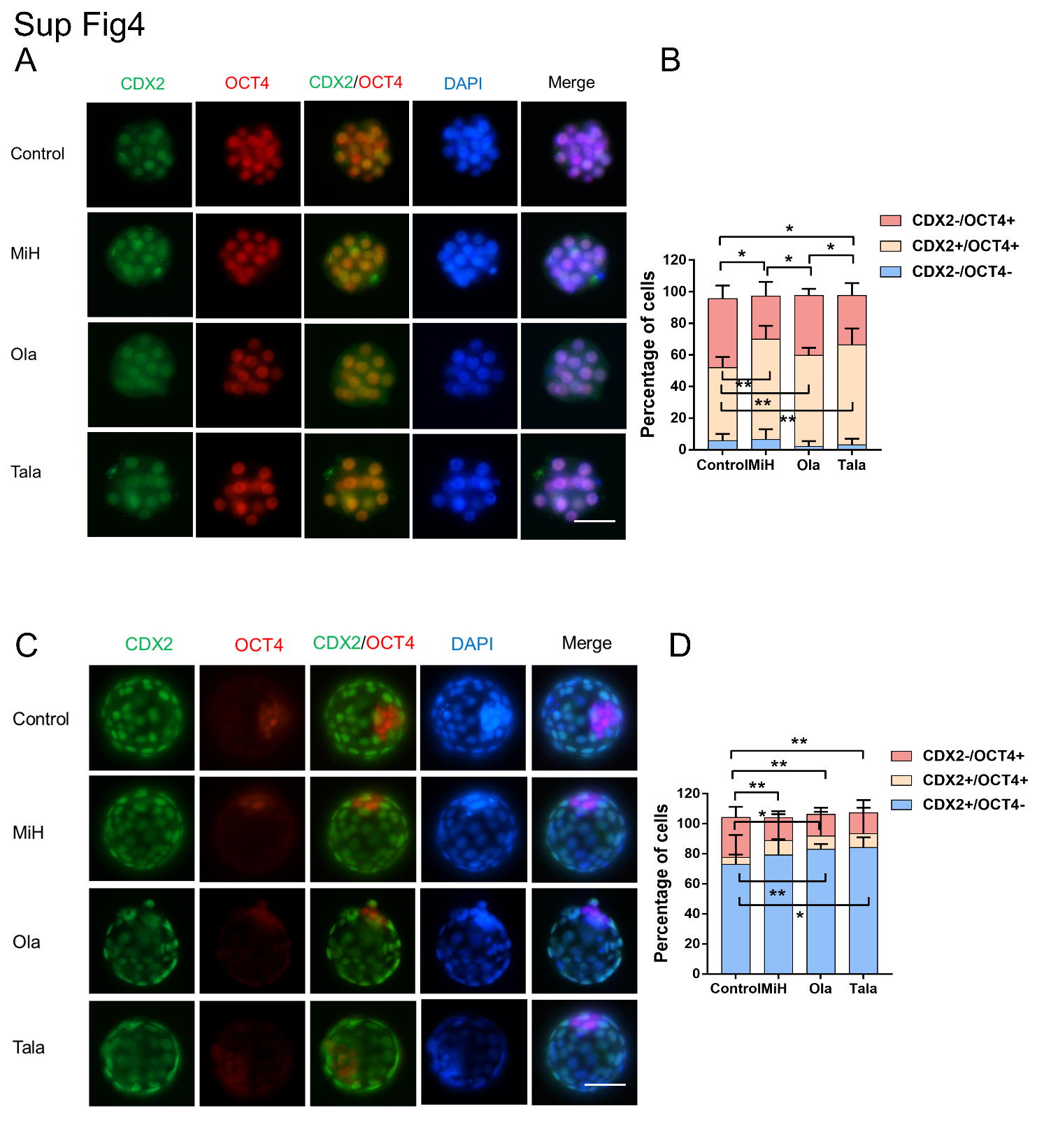


**Supplementary Figure 4. PARP1 inhibitors affected ICM and TE specification of mouse preimplantation embryos.** (A-B) Immunofluorescent staining of morula and counts for CDX2-/OCT4+, CDX2+/OCT4+ and CDX2-/OCT4- cells portions of each embryo. N (Control)=11, n (MiH)=11, n (Ola)=11, n (Tala)=10. (C-D) Immunostaining of expanded and hatching embryos and counts for CDX2-/OCT4+, CDX2+/OCT4+ and CDX2+/OCT4- cells portions of each blastocyst at 100 h. Bars=50 μm. N (Control)=11, n (MiH)=11, n (Ola)=11, n (Tala)=11. Data are presented as mean ± SD in three independent experiments. Student’s t tests were used for statistical analysis. ∗, 0.01<P <0.05; ∗∗, P<0.01; no labeling indicates no significant difference.

**
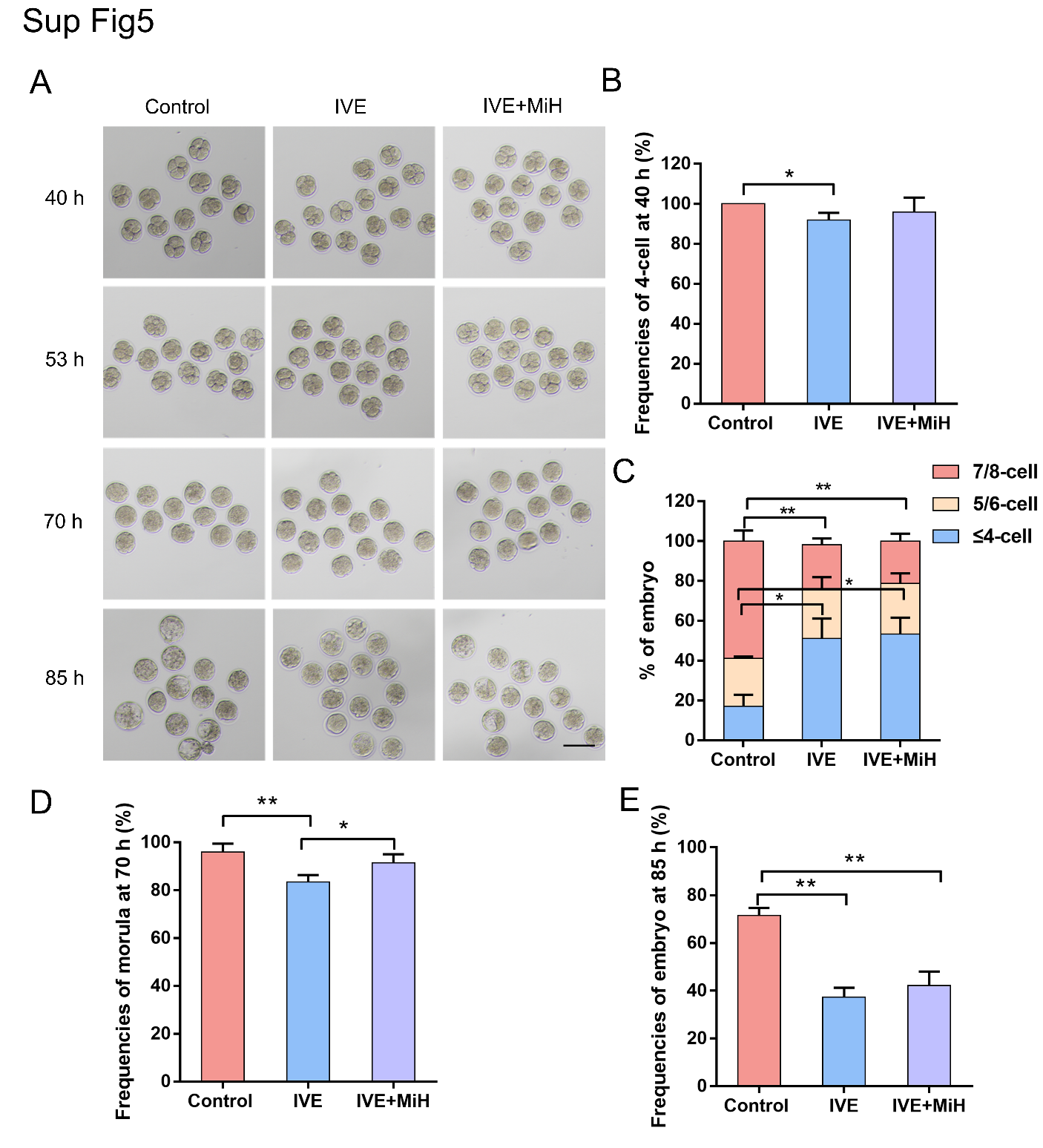
**

**Supplementary Figure 5. The exposure to external environment impaired embryo developmental potential.** (A) Representative images of embryos at 40 h, 53 h, 70 h and 85 h respectively after fertilization. Bar=100 μm. (B-E) Frequencies of embryos at different time points. 46, 49, 47 embryos were totally counted in Control, IVE and IVE+MiH groups. Data are presented as mean ± SD in three independent experiments. Student’s t tests were used for statistical analysis. ∗, 0.01<P <0.05; ∗∗, P<0.01; no labeling indicates no significant difference.

**
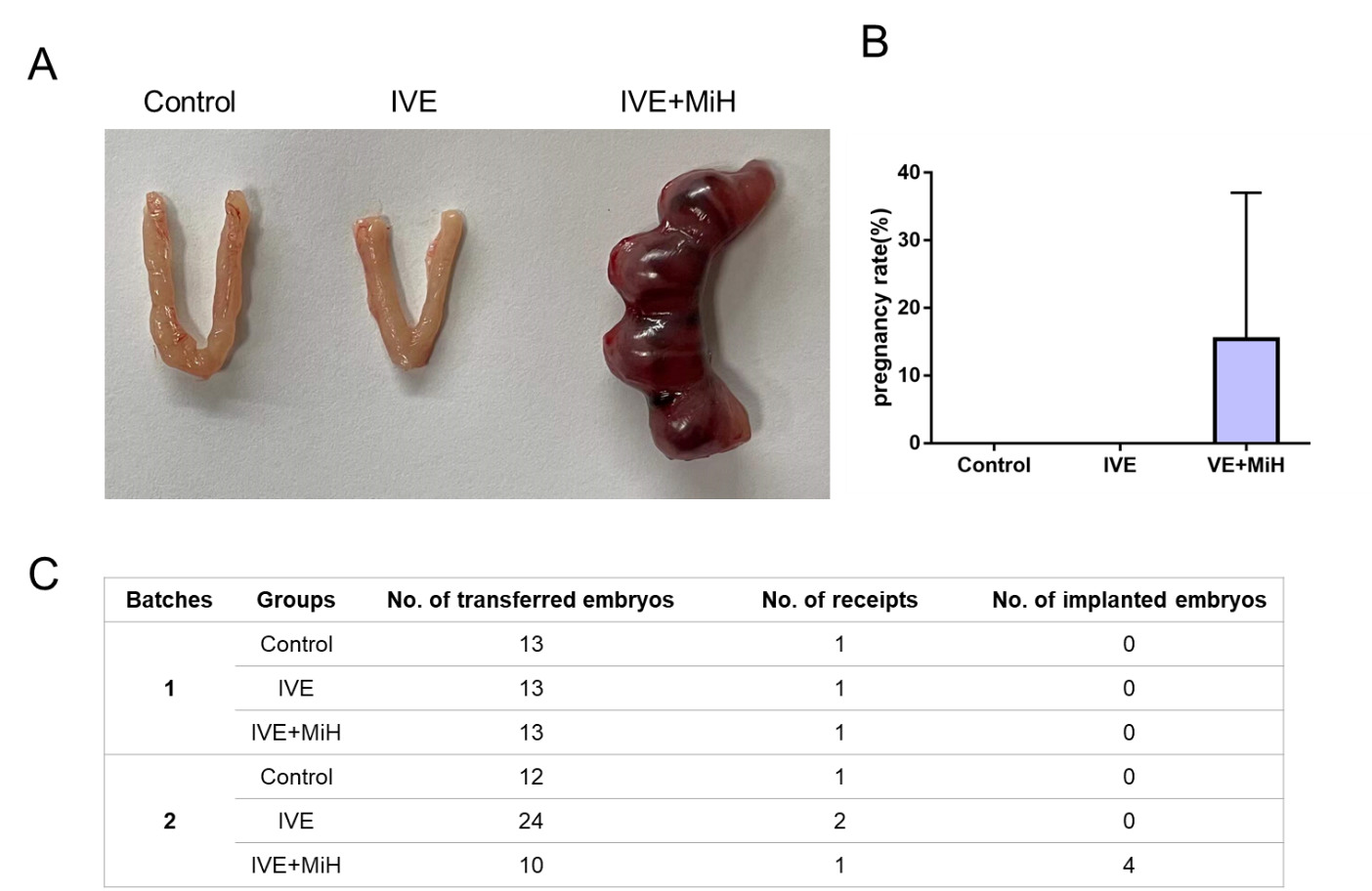
**

**Supplementary Figure 6. Representative image, pregnancy rate and statstics of a table of implanted embryos on day 9 after being transferred. n (Control)=25, n (IVE)=37, n (IVE+MiH)=23.**

**
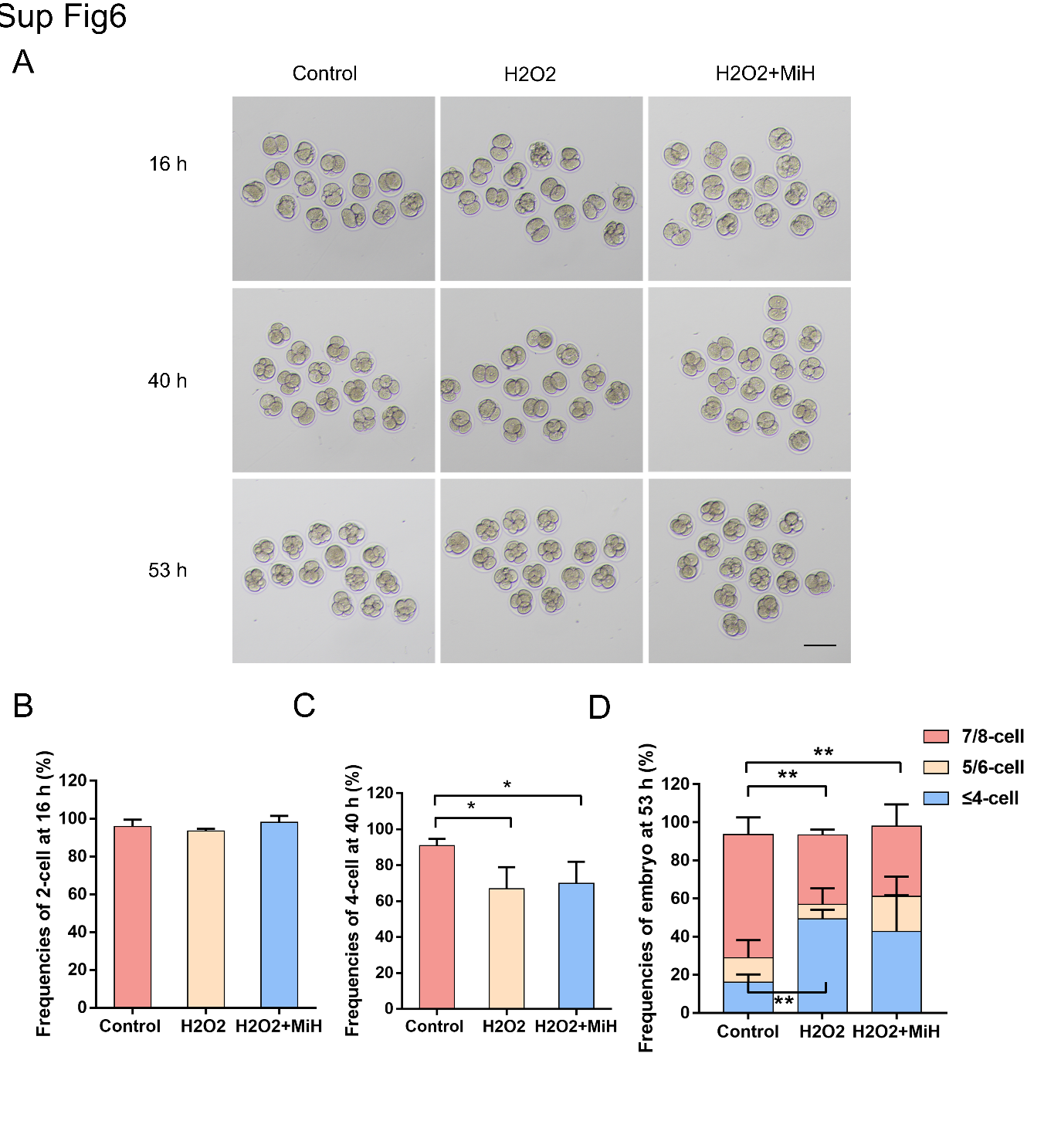
**

**Supplementary Figure 7. The mouse zygotes which had suffered from H2O2 developed slowly since 40 h.** (A) Representative images of embryos observed at 16 h, 40 h and 53 h respectively after fertilization. Bar=100 μm. (B-D) Frequencies of embryos at each stage in three groups. 44, 47, 45 embryos were totally counted in Control, H2O2- and H2O2+MiH-treated groups, respectively. Data are presented as mean ± SD in three independent experiments. Student’s t tests were used for statistical analysis. ∗, 0.01<P <0.05; ∗∗, P<0.01; no labeling indicates no significant difference.

**2. Supplementary Table 1. The components of KSOM.**

| Component | mg/100ml |
| --- | --- |
| EDTA  Sodium-Pyruvate  Glucose  KH2PO4  MgSO4 X 7H2O  Streptomycin  Penicillin G  Glutamine  KCl  NaHCO3  NaCl  Sodium lactate 60%  Ess.-AminoacidX50  Non-ess. AminoacidX100  Phenol red  CaCl2 X 2H2O  BSA | 0.38  2.2  3.6  4.75  4.95  5  6.3  14.5  18.5  210  559.5  174 ul  1000 ul  500 ul  0.1  25  100 |
